# Comparison of the Efficacy of Ultrasound-Guided Serratus Anterior Plane Block Versus Erector Spinae Plane Block for Postoperative Analgesia After Modified Radical Mastectomy: A Randomised Controlled Trial

**DOI:** 10.5152/TJAR.2022.21127

**Published:** 2022-12-01

**Authors:** Deepti Ahuja, Vinod Kumar, Nishkarsh Gupta, Sachidanand Jee Bharati, Rakesh Garg, Seema Mishra, Maroof Ahmad Khan, Sushma Bhatnagar

**Affiliations:** 1 Department of Onco-Anaesthesia and Palliative Medicine, Dr. B. R. Ambedkar Institute Rotary Cancer Hospital, All India Institute of Medical Sciences, New Delhi, India; 2 Department of Bio-Statistics, All India Institute of Medical Sciences, Ansari Nagar, New Delhi, India

**Keywords:** Erector spinae plane block, modified radical mastectomy, pain, regional anaesthesia, serratus anterior plane block

## Abstract

**Objective:**

Several interfascial interfacial plane blocks have been described in patients undergoing modified radical mastectomy. We conducted this study to evaluate the analgesic efficacy of ultrasound-guided serratus anterior plane block and erector spinae plane block in patients undergoing modified radical mastectomy.

**Methods:**

Totally, 80 female patients (18-70 years) undergoing modified radical mastectomy were randomised into 2 groups of 40 each and were given ultrasound-guided serratus anterior plane block or erector spinae plane block with 0.4 mL kg^−1^ of 0.375% ropivacaine in this prospective double-blind control trial. The groups were compared for the time to request of first dose of rescue analgesic, requirement of rescue analgesics, and patient satisfaction score.

**Results:**

: The time to request of the first rescue analgesia was comparable in both groups (*P*  = .056). Postoperative pain scores at rest at 0 minute were significantly lower in serratus anterior plane group as compared to erector spinae plane group (*P*  = .03). The intraoperative fentanyl requirement and postoperative diclofenac and tramadol requirements were comparable between the 2 groups. The number of patients requiring rescue doses of fentanyl intraoperatively and rescue analgesics postoperatively was similar in both groups. The mean patient satisfaction score was also comparable in both groups.

**Conclusion:**

Ultrasound-guided serratus anterior plane block and erector spinae plane block have comparable postoperative analgesic efficacy after modified radical mastectomy.

**Main Points**

- Serratus anterior plane (SAP) block and erector spinae plane (ESP) block are being used to provide analgesia for breast cancer surgery.
- This study compared the effect of ultrasound-guided SAP block and ESP block on time to request of the first rescue analgesia in patients undergoing modified radical mastectomy (MRM).
- Ultrasound-guided SAP block and ESP block have comparable postoperative analgesic efficacy after MRM.

## Introduction

Modified radical mastectomy (MRM) is associated with significant pain during the immediate postoperative period.^1^ Uncontrolled, acute postoperative pain can lead to an increased surgical stress response, cardiac and pulmonary complications, opioid-related adverse events, and longer stay in the postanaesthesia care unit (PACU). The presence of poorly controlled acute postoperative pain is a risk factor for the development of chronic postsurgical pain.^2^ The chronic pain after breast cancer surgery may occur in 20%-50% of patients and influences the activities of daily living resulting in a poor quality of life.^3^ Thoracic epidural^4^ and paravertebral blocks^5^ are often considered inordinate for minimally invasive breast surgery due to associated complications. Hence, various ultrasound-guided thoracic interfascial plane blocks including pectoral nerve block type 1 (PECS I), modified PECS block (PECS II), serratus anterior plane (SAP) block, and erector spinae plane (ESP) block are gaining acceptance as analgesic techniques in patients undergoing breast cancer surgery.^6^ These blocks require deposition of local anaesthetic in an interfascial plane through which peripheral nerves travel.^6^ Serratus anterior plane block, first described by Blanco et al^7^ involves the injection of local anaesthetic in 1 of the 2 fascial planes, that is, superficial and deep to serratus anterior muscle at the level of the fifth rib in midaxillary line. The SAP block targets the lateral cutaneous branches of the thoracic intercostal nerves.^8^ The deep SAP block was found to have similar analgesic efficacy and technically easier and safer to perform as compared to the superficial SAP block.^9^ Erector spinae plane block is another novel interfascial plane block, first described by Forero et al.^10^ It involves deposition of local anaesthetic between erector spinae muscle and transverse process of T5 vertebrae and targets both dorsal and ventral rami of thoracic spinal nerves. The analgesic efficacy of ultrasound-guided SAP block and ESP block is better than conventional opioid-based analgesia^11,12^ and comparable with thoracic PVB^13,14^ in patients undergoing breast cancer surgery. Furthermore, ESP block has the additional advantage of avoiding the deposition of local anaesthetic at the surgical site in the axillary area. However, literature comparing the 2 blocks in patients undergoing breast cancer surgery is limited. This prospective randomised double-blind controlled trial compared the analgesic efficacy of ultrasound-guided SAP block with ESP block in patients undergoing MRM.

## Methods

This study was carried out at a tertiary care centre from October 2018 to December 2019. The study protocol was approved by the All India Institute of Medical Sciences Ethics Committee (IECPG-12/28.06.2018) and was registered prospectively in Clinical Trial Registry, India (www.ctri.nic.in) with identification number CTRI/2018/07/014913, dated: July 07, 2018. Totally, 80 female patients aged 18-70 years, belonging to the American Society of Anaesthesiologists (ASA) physical status I-III, undergoing MRM, and who gave written informed consent were included in the study. Patients with infection at the site of injection, severe chest wall deformity, history suggestive of coagulopathy, presence of severe heart disease (NYHA classification ≥3), renal or hepatic disorder, allergic to any drug used in the study, and patients who refused to give written informed consent were excluded from the study.

The patients were randomly allocated to 1 of the 2 groups using computer-generated randomisation table. Allotment concealment was done with the sequentially numbered opaque-sealed envelope technique, which was opened after recruitment in the preoperative holding area. The patients in group SAP received ultrasound-guided SAP block with 0.375% ropivacaine (0.4 mL kg^−1^) with general anaesthesia and the patients in group ESP received ultrasound-guided ESP block with 0.375% ropivacaine (0.4 mL kg^−1^) with general anaesthesia.

### Anaesthetic Technique

Preoperative assessment of patients was done 1 day prior to surgery. After shifting patients to the preoperative holding area, standard ASA monitors were applied, and baseline readings were noted. Block was administered by anaesthesiologist who was not involved in further management of case before the induction of general anaesthesia. Heart rate (HR), electrocardiogram (ECG), oxygen saturation (SpO_2_), systolic blood pressure (SBP), diastolic blood pressure (DBP), and mean arterial pressure (MAP) were continuously monitored. Number of attempts and the time taken to perform the block were recorded. Time to perform the block was calculated from the beginning of ultrasound scanning to the completion of deposition of the local anaesthetic in the interfascial plane. Block failure was defined as the inability of the anaesthesiologist to visualise the placement of the needle tip and spread of local anaesthetic in the correct interfascial plane.

### Procedure Done in Serratus Anterior Plane group

**Figure 1.**
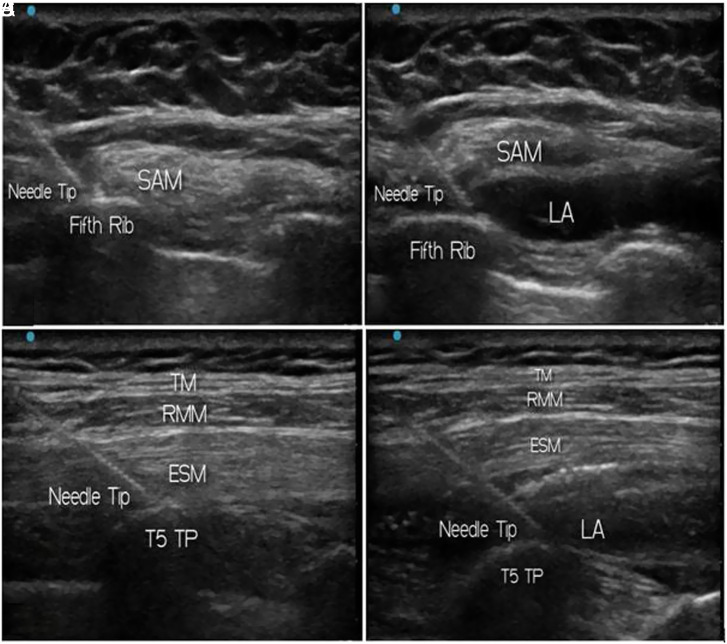
(A, B) Depicting serratus anterior plane block; (C, D) depicting erector spinae plane block. SAM, serratus anterior muscle; LA, local anaesthetic; TM, trapezius muscle; RMM, rhomboid major muscle; ESM, erector spinae muscle; TP, transverse process.

Serratus anterior plane block was administered to patient in the supine position with ipsilateral arm abducted to 90°. Under aseptic precautions, linear high-frequency (6-15 MHz) ultrasound probe (Edge II; SonoSite, Inc., Bothell, Wash, USA) was placed over the midclavicular region in the sagittal plane. Ribs were counted inferiorly and laterally until the fifth rib was identified in midaxillary line. Latissimus dorsi, teres major, and serratus anterior muscles were identified overlying the fifth rib. The intended puncture site was infiltrated with 2 mL of 2% lignocaine**, **and using ultrasound-guided in-plane approach, 22 G echogenic, 80-mm long block needle (SonoTAP; Pajunk) was introduced in caudal to cranial direction until the tip was placed between the serratus anterior muscle and external intercostal muscle. After confirming the placement of the needle in interfascial plane by hydrodissection with 2 mL normal saline (Figure 1A) and negative aspiration for blood, 0.4 mL kg^−1^ of 0.375% ropivacaine (Ropin 0.75%; Neon) with a maximum volume of 30 mL was injected (Figure 1B).

### Procedure Done in Erector Spinae Plane Group

Erector spinae plane block was administered to patient in a lateral position (depending on the side of surgery). Under aseptic precautions, a linear high frequency (6-15 MHz) ultrasound probe (Edge II; SonoSite, Inc., Bothell, Wash, USA) was placed in a longitudinal orientation, 3 cm lateral to T5 spinous process. Trapezius, rhomboid major, and erector spinae muscles were identified as superficial to hyperechoic transverse process. The intended puncture site was infiltrated with 2 mL of 2% lignocaine, and using ultrasound-guided in-plane approach, 22 G echogenic, 80-mm long block needle (SonoTAP; Pajunk) was introduced from cranial to caudal direction until the tip was placed between the erector spinae muscle and transverse process. After confirming the placement of the needle in interfascial plane by hydrodissection with 2 mL normal saline (Figure 1C) and negative aspiration for blood, 0.4 mL kg^−1^ of 0.375% ropivacaine (Ropin 0.75%; Neon) with a maximum volume of 30 mL was injected (Figure 1D).

### Procedure Common to Both Groups

After administering the block, patients were monitored for complications including intravascular injection, pneumothorax, hypotension, and bradycardia. Patients received general anaesthesia in standardised manner by anaesthesiologist who was blinded to group allotment. Anaesthesia induction was done using intravenous fentanyl 2 µg kg^−1^, propofol 2 mg kg^−1^, and atracurium 0.5 mg kg^−1^ followed by the insertion of appropriate size proseal laryngeal mask airway (PLMA). Intravenous dexamethasone (0.1 mg kg^−1^) and paracetamol (15 mg kg^−1^) were administered immediately after induction. Anaesthesia was maintained with desflurane with mixture of O_2_ (50%) and air (50%) to maintain minimum alveolar concentration (MAC) between 0.8 and 1 and intermittent boluses of atracurium**. **Patients were ventilated to maintain end-tidal concentration of carbon dioxide (EtCO_2_) between 35 and 45 mmHg. Intraoperatively, HR, SBP, DBP, MAP, ECG, SpO_2_, temperature, EtCO_2,_ and MAC were monitored at intervals of 5 minutes for the first half an hour and then every 10 minutes till completion of surgery. Intravenous fentanyl (0.5 µg kg^−1^) was given if HR or SBP increased by ≥20% of baseline. Rescue dose of intraoperative fentanyl given was recorded. Hypotension was defined as a decrease in MAP by ≥20% of baseline value and was treated with 250 mL of fluid bolus and intravenous mephenteramine of 6 mg. If HR was decreased by ≥20% of baseline value or was ≤60/minute, intravenous atropine of 0.6 mg was given. After completion of surgery, residual neuromuscular block was reversed and PLMA was removed. Patients were shifted to PACU for monitoring and pain assessment.

### Postoperative Monitoring

Pain scores were recorded at rest and during arm abduction using 11-point Numeric Rating Scale (NRS: 0 = no pain; 10 = worst imaginable pain) scores at 0 minute, 30 minutes, 1, 2, 4, 8, 12, 18, and 24 hours postoperatively. A standard algorithm was used for postoperative pain management, which involved the administration of an intravenous infusion of paracetamol of 15 mg kg^−1^ immediately after induction and repeated every 8 hours for the first 24 hours. On assessment, if NRS was ≥3 at rest or arm abduction, first rescue analgesia, intravenous diclofenac sodium (1.5 mg kg^−1^) was administered; not repeated within 12 hours of the first dose. Patients were reassessed after 30 minutes; if NRS ≥3 persisted, a second rescue analgesic intravenous tramadol hydrochloride (1 mg kg^−1^) was administered. Duration of analgesia was defined as the time from extubation to the time to reach NRS ≥3, at which patients received the first rescue analgesia and was recorded.

Postoperative nausea and vomiting (PONV grade: 0 = no nausea and vomiting, 1 = nausea without vomiting, 2 = nausea with vomiting <3 episodes, 3 = nausea with vomiting ≥3 episodes) were recorded at the same time intervals. If the patient complained of persistent nausea or vomiting, intravenous ondansetron of 0.1 mg kg^−1^ was administered and recorded. Hemodynamic parameters and side effects like respiratory depression and pruritus were recorded. Patient satisfaction was evaluated using 7-point Likert scale (1 = extremely dissatisfied; 7 = extremely satisfied) at 24 hours postoperatively. Assessments were done by anaesthesiologist not involved in the administration of block or intraoperative management of patients.

### Outcome Measures

Primary outcome was the time to request of the first rescue analgesia. Secondary outcomes were total consumption of rescue doses of intravenous fentanyl intraoperatively, intravenous tramadol hydrochloride and diclofenac sodium, and patient satisfaction score, at 24 hours postoperatively.

### Statistical Analysis

The sample size calculation was based on a preliminary pilot study by Gupta et al^13^ who compared the analgesic efficacy of ultrasound-guided PVB versus SAP block and reported the time to request of the first rescue analgesia after SAP block as 234 ± 60 minutes. We anticipated an equivalence margin of 35 minutes for ESP block. The sample size for 80% power with 5% level of significance was calculated as 40 patients in each group. So , the total sample size for our study was 80.

We used statistical software International Business Machines Statistical Package for the Social Sciences version 24 (IBM Corp., Armonk, NY, USA) for statistical analysis. The quantitative variables were compared using unpaired *t*-test or Mann–Whitney test (if data sets are not normally distributed between 2 groups). The qualitative variables were compared using chi-square test or Fisher’s exact test. *P* <.05 was considered to be statistically significant.

## Results

**Figure 2.**
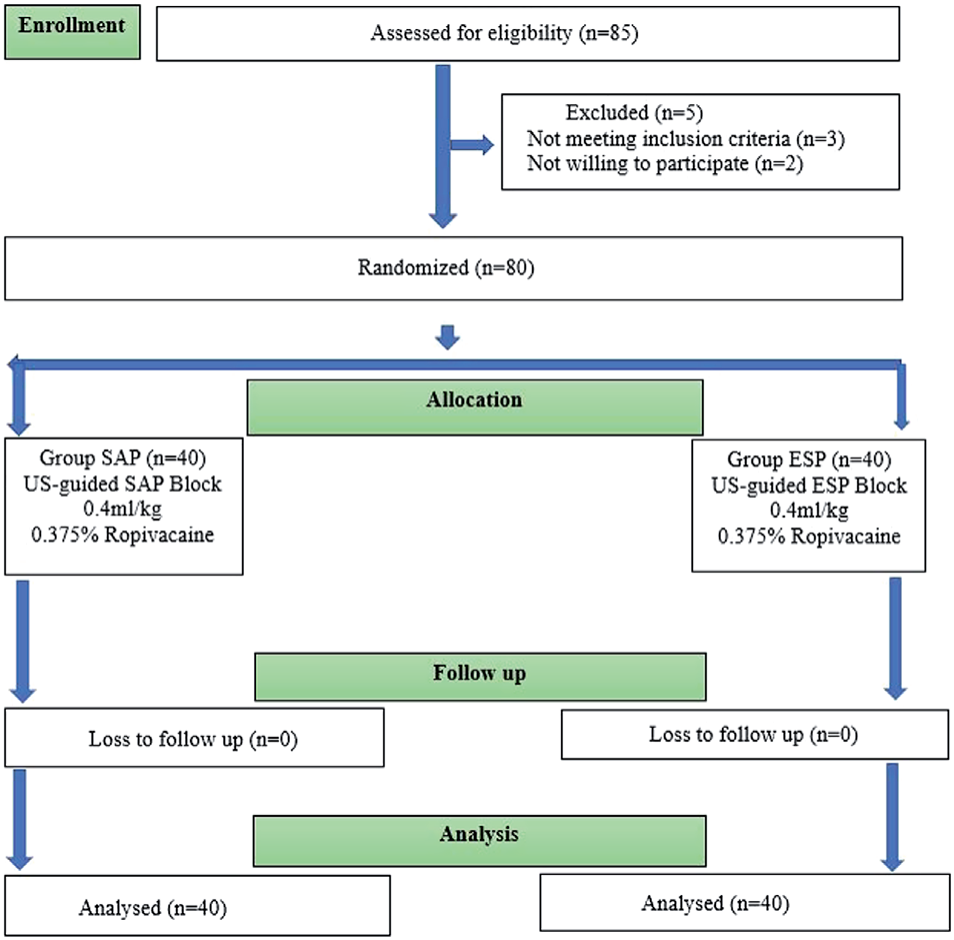
The Consolidated Standards of Reporting Trials diagram.

The Consolidated Standards of Reporting Trials flow diagram for this trial is shown in Figure 2. Totally, 85 patients were assessed for enrolment in the study, with 80 patients meeting the inclusion criteria and were randomised into 2 groups of 40 each.

The demographic data, ASA physical status, duration of surgery, and number of attempts taken to perform the block were similar in both groups. However, time taken to perform ESP block was significantly more as compared to SAP block (*P*  =.035) (Table 1).

The median (interquartile range) time to request of the first rescue analgesia was 252.50 (221.25-300) minutes in SAP group and 215 (170-260) minutes in ESP group (Table 2). The time to request of first rescue analgesia was comparable in both groups (*P* = .056). The number of patients requiring rescue analgesia 24 hours postoperatively was 55% (22/40) in SAP group and 72.5% (29/40) in ESP group (*P*  = .106).

The rescue dose of fentanyl (µg) administered intraoperatively was comparable in both groups (SAP vs. ESP: 39.8 ± 20.4 vs. 33.7 ± 7.7; *P*  = .500) (Table 2). The number of patients requiring rescue doses of fentanyl intraoperatively was also comparable in both groups (SAP vs. ESP: 22.5% (9/40) vs. 15% (6/40); *P*  = .390) (Table 2).

Postoperative pain scores at rest at 0 minute was significantly less in SAP group as compared to ESP group (*P* = .034) (Table 3). The pain scores at rest at other time points and during arm abduction at all time points were comparable in both groups (Table 3). The postoperative 24-hour requirement of diclofenac (mg) and tramadol (mg) was less in SAP group (80.6 ± 23.05; 56.7 ± 8.4) as compared to ESP group (91.3 ± 30.8; 75.2 ± 19.5), but the difference was not statistically significant (*P*  = .179; *P*  = .111) (Table 2). The number of patients requiring rescue analgesia postoperatively was also comparable in both the groups (Table 2).

The incidence of PONV and the requirement of ondansetron were similar in both groups (Table 2). The mean patient satisfaction score was higher in SAP group (6.1 ± 0.87) as compared to ESP group (5.7 ± 0.76), but the difference was not statistically significant (*P*  = .080) (Table 2).

The hemodynamic variables (HR, SBP, DBP) were comparable in both groups throughout the period of study. None of the patients developed technique-related complications like intravascular injection, pneumothorax, and local anaesthetic systemic toxicity. Adverse effects like pruritus and respiratory depression were not seen in any patient postoperatively.

## Discussion

This prospective randomised controlled trial showed that ultrasound-guided SAP block and ESP block have comparable postoperative analgesic efficacy in patients undergoing MRM. The time to request of the first rescue analgesic in the postoperative period, postoperative pain scores at rest (except at 0 minute) and movement, intraoperative fentanyl requirement and postoperative analgesic requirement, and mean patient satisfaction score were comparable in both groups. Both fascial plane blocks provided hemodynamical stability with minimal side effects. However, the time taken to perform SAP block was significantly shorter than ESP block.

The breast tissue is innervated by the anterior and lateral cutaneous branches of T1-T6 intercostal nerves, supraclavicular branches of the superficial cervical plexus, medial and lateral pectoral nerves, and thoracodorsal and long thoracic nerves.^15^ Regional anaesthesia technique provides adequate analgesia, suppresses surgical stress response, reduces the requirement of opioids, and may prevent cancer recurrence.^16,17^

In superficial SAP block, the spread of local anaesthetic leads to disruption of axillary tissue planes, blockade of long thoracic and thoracodorsal nerves, difficulty in the identification and preservation of nerves intraoperatively, and needling through potential metastatic lymph nodes, increasing the chances of tumor seeding.^9^ The deep SAP block is technically easier and safer to perform as it uses the rib as the end point of postoperative analgesia and provides similar analgesia as superficial SAP block.^9^ So, we preferred to use a deep SAP block in our study. Since its introduction by Forero et al.^10^ ESP block has been acclaimed as “magic bullet” for postoperative analgesia after thoracoabdominal surgeries.^18^

The difference in the duration of analgesia after various blocks is due to different planes of drug deposition and the nerves anaesthetised.^15,19^ The differences observed in the duration of analgesia even after administration of standardised weight-based volume and concentration of local anaesthetic in a block can be attributed to the variation in structure and function of deep fascia and interfascial journey of somatic and sympathetic nerves.^20^ The time to request of the first rescue analgesia found in this study was comparable with previous studies.^13,21-23^

We found that the rescue dose of fentanyl and the total dose of fentanyl administered intraoperatively was comparable in both groups. The results of our study were found to be comparable with previous studies.^13,23-25^ Few studies have reported the consumption of lower doses of fentanyl in patients undergoing mastectomy after the administration of ESP block.^26,27^ The smaller doses are attributed to lower doses of fentanyl (1 µg kg^−1^) given at the time of induction and shorter mean duration of surgery (78.3 minutes) as compared to our study.^26,27^

In this study, the number of patients requiring rescue analgesia (diclofenac sodium or tramadol) in the first 24 hours postoperative period was also comparable in both groups. These results were comparable with previous studies.^11,12,25,27^ Altıparmak et al^26^ administered ESP block with 20 mL of 0.25% bupivacaine and used tramadol patient-controlled analgesia (PCA) in the postoperative period and reported tramadol consumption of 196 ± 27.03 mg in patients undergoing radical mastectomy. They reported that 50% of patients required rescue analgesia with morphine (intravenous morphine of 4 mg if NRS was ≥4 during coughing) postoperatively despite receiving a high dose of tramadol. In our study, the reduced requirement of tramadol may be due to opioid-sparing effect of non-steroidal anti-inflammatory drugs (NSAIDS; acetaminophen and diclofenac sodium) that were given before initiating opioids. The postoperative median NRS at rest (except at 0 minute) and movement was comparable in both groups at all other time points and the results were comparable with the previous studies.^28,29,30^

The incidence of PONV and the requirement of ondansetron were found to be similar in both groups. The results of our study were similar to the study done by Gupta et al.^13^ Some studies have reported higher incidence of PONV probably because of nitrous oxide used by them for the maintenance of anaesthesia and intravenous tramadol of 100 mg given for postoperative analgesia at the completion of surgery in all patients,^12,14^ whereas, in our study, we had used air : oxygen mixture and given tramadol in patients on demand after NSAIDs failed to relieve pain. This led to high satisfaction scores in all patients of both groups like in previous studies.^28-29^

None of the patients in either group developed technique-related complications like hemodynamic instability, intravascular injection, pneumothorax, local anaesthetic systemic toxicity, and adverse effects like pruritus and respiratory depression.

A recent randomised controlled trial by Finnerty et al^8^ reported that ESP block provided superior quality of recovery at 24 hours postoperatively, reduced morbidity, and better analgesia after minimally invasive thoracic surgery compared to SAP block. The contrasting results as compared to our results can be explained by the different surgical populations studied. The acute pain after MRM is determined mainly by the somatic aspect, whereas, post-thoracotomy pain includes somatic and visceral components along with shoulder pain. The local anaesthetic administered in ESP block potentially spreads to the thoracic paravertebral space and provides better pain control than the SAP block in thoracic surgery.

This trial showed that both fascial plane blocks have comparable analgesic efficacy in patients undergoing MRM. One of the strengths of our study is the use of standardised weight-based volume and concentration of local anaesthetic, providing an equitable comparison of the analgesic efficacy of ultrasound-guided SAP and ESP block in patients undergoing MRM.

We did not use PCA device in the postoperative period which could have better quantified opioid requirements. The lack of a control group is another limitation of the study, as it would strengthen the results of our study. The effect of regional anaesthesia technique on chronic pain and cancer recurrence could not be assessed as follow-up of patients was done for a limited time.

## Conclusion

Ultrasound-guided SAP block and ESP block have comparable postoperative analgesic efficacy after MRM. The safety and efficacy of these blocks underpin their routine use as part of multimodal analgesia protocols in patients undergoing MRM.

## Figures and Tables

**Table 1.** Comparison of Demographic Variables and Block Procedure Data in 2 Groups

Variables	Group SAP (n = 40)	Group ESP (n = 40)	*P*
Age (years)	45.9 ± 10.0	49.6 ± 11.5	.126
Weight (kg)	59.8 ± 9.4	61.9 ± 11.2	.377
Height (cm)	153.6 ± 8.6	152.6 ± 5.7	.575
BMI (kg m^-2^)	25.5 ± 3.6	26.5 ± 4.4	.314
ASA physical status (I/II/III)	23/17/0	15/24/1	.117
Attempts taken to perform block (1/2/3)	39/1/0	37/2/1	.615
Time taken to perform block (minutes)	11.1 ± 3.8	13.1 ± 4.4	.035
Duration of surgery (minutes)	125.6 ± 33.2	126.8 ± 34.9	.870

Data are expressed as mean ± SD or numbers.

ASA, American Society of Anaesthesiologists; BMI, body mass index; SD, standard deviation; SAP, serratus anterior plane; ESP, erector spinae plane.

**Table 2.** Comparison of Quality of Analgesia and Adverse Effects in 2 Groups in Postoperative 24 Hours

Variable	Group SAP (n = 40)	Group ESP (n = 40)	*P*
Duration of analgesia (minutes)	252.50 (221.25-300)	215 (170-260)	.056
Rescue dose of intraoperative fentanyl (µg)	39.8 ± 20.4	33.7 ± 7.7	.500
Total dose of intraoperative fentanyl (µg)	128.77 ± 29.19	129.22 ± 29.09	.945
Patients requiring rescue doses of fentanyl intraoperatively (n, %)	9 (22.5%)	6 (15%)	.390
Rescue dose of diclofenac sodium (mg)	80.6 ± 23.05	91.3 ± 30.8	.179
Rescue dose of tramadol (mg)	56.7 ± 8.4	75.2 ± 19.5	.111
Patients requiring rescue analgesics postoperatively (n, %)	22 (55%)	29 (72.5%)	.106
Incidence of postoperative nausea (n, %)	7 (17.5%)	8 (20%)	1.000
Incidence of postoperative vomiting (n, %)	3 (7.5%)	3 (7.5%)	1.000
Postoperative requirement of ondansetron (mg)	6.5 ± 1.0	6.5 ± 0.92	.904
Patient satisfaction score	6.1 ± 0.87	5.7 ± 0.76	.080

Data are expressed as median (interquartile range), mean ± SD, and numbers (%).

SD, standard deviation; SAP, serratus anterior plane;ESP, erector spinae plane.

**Table 3.** Post-operative Numeric Rating Scores (NRS) at Rest and During Arm Abduction

Time	Group SAP		Group ESP		*P*	
	Rest	Arm Abduction	Rest	Arm Abduction	Rest	Arm Abduction
0 minute	0.5 (0-1)	2 (0.25-2)	1 (0-5.75)	2 (0-7.75)	.034	.093
30 minutes	1 (0-1)	2 (1-2)	1 (0-1)	2 (2-2)	.555	.601
1 hour	1 (0.25-1.75)	2 (1.25-2)	1 (1-1)	2 (2-2)	.685	.257
2 hours	1 (0-1)	2 (1.25-2)	1 1-1)	2 (2-2)	.280	.151
4 hours	1 (0-1)	2 (1-2)	1 (0-1)	2 (1-2	.654	.720
8 hours	1 (0-1)	2 (1-2)	1 (0-1)	2 (1-2)	.385	.340
12 hours	0 (0-1)	1 (1-2)	0.5 (0-1)	1.5 (1-2)	.353	.319
18 hours	0 (0-1)	1 (1-2)	0 (0-1)	1 (1-2)	.839	.970
24 hours	0 (0-1)	1 (1-1.75)	0 (0-0.75)	1 (1-1.75)	.801	.741

Data are expressed as median (interquartile range).

SAP, serratus anterior plane; ESP, erector spinae plane.
